# β-Ga_2_O_3_ MOSFETs electrical characteristic study of various etching depths grown on sapphire substrate by MOCVD

**DOI:** 10.1186/s11671-023-03867-9

**Published:** 2023-06-27

**Authors:** Chan-Hung Lu, Fu-Gow Tarntair, Yu-Cheng Kao, Niall Tumilty, Jia-Min Shieh, Shao-Hui Hsu, Ching-Lien Hsiao, Ray-Hua Horng

**Affiliations:** 1 Institute of Electronics, National Yang Ming Chiao Tung University, Hsinchu, 30010 Taiwan, ROC; 2 Department of Materials Science and Engineering, National Chung Hsing University, Taichung, 40227 Taiwan, ROC; 3 International College of Semiconductor Technology, National Yang Ming Chiao Tung University, Hsinchu, 30010 Taiwan, ROC; 4 National Applied Research Laboratories, Taiwan Semiconductor Research Institute (TSRI), Hsinchu, 30091 Taiwan, ROC; 5 Thin Film Physics Division, Department of Physics, Chemistry and Biology (IFM), Linköping University, 581 83 Linköping, Sweden

**Keywords:** High power MOSFETs, Enhancement mode, β-Ga_2_O_3_, Recessed gate, MOCVD

## Abstract

β-Ga_2_O_3_ thin films with both a 45 nm Si-doped conductive epilayer and unintentionally doped epilayer were grown on c-plane sapphire substrate by metalorganic chemical vapor deposition. β-Ga_2_O_3_ based metal–oxide–semiconductor field-effect transistors (MOSFETs) were fabricated with gate recess depths of 20 nm and 40 nm (it indicated gate depth with 70 nm and 50 nm, respective), respectively, and without said recessing process. The conductivity of β-Ga_2_O_3_ epilayers was improved through low in situ doping using a tetraethoxysilane precursor to increase MOSFET forward current density. After recessing, MOSFET operation was transferred from depletion to enhanced mode. In this study, the maximum breakdown voltage of the recessed 40 nm transistor was 770 V. The etching depth of a recessed-gate device demonstrates its influence on device electrical performance.

## Introduction

β-Ga_2_O_3_ is emerging as the potential high-power device candidate for next generation applications owing to its ultra-wide bandgap of 4.8 eV [1]. It is known that β-Ga_2_O_3_ possesses a critical electric field value of 8 MV/cm considerably higher than more established power semiconductor materials such as 4H-SiC (2.5 MV/cm) and GaN (3.3 MV/cm) [2]. From Baliga’s figure of merit (BFOM) the trade-off between on-resistance and breakdown voltage demonstrates that β-Ga_2_O_3_ (3214) has greater potential for high power applications compared to 4H-SiC (317) and GaN (846) [3]. An on-going and critical problem for both SiC and GaN-based devices is the higher crystal growth cost. For β-Ga_2_O_3_, costs are an order of magnitude lower and films can be grown hetero-epitaxially by different methods [4, 5]. In this context, high quality heteroepitaxil β-Ga_2_O_3_ layers can be grown by metalorganic chemical vapor deposition (MOCVD) on sapphire which is clearly in reducing growth cost [5].

These superlative material characteristics permit β-Ga_2_O_3_ to be employed for many electrical devices, such as metal–oxide–semiconductor field-effect transistors (MOSFET) [6–10], metal–semiconductor field-effect transistors (MESFET) [11], and Schottky barrier diodes [12]. In addition, to increasing conductivity, most devices are grown homoepitaxially and doped by Si-ion implantation forming a shallow donor [13]. Furthermore, there are many studies on both depletion-mode (D-mode) MOSFETs using Ga_2_O_3_ epilayers on bulk Ga_2_O_3_ substrates and Ga_2_O_3_ grown on sapphire substrates combined with Si-ion implantation technologies [6–8]. However, the lack of a p-type-based epitaxial substrate [14] leads to fewer enhancement-mode (E-mode) MOSFET published examples and clearly needs to be developed using other special techniques, which is the purpose of this work. Recently, a number of E-mode MOSFETs have been reported, such as Sn-doped Ga_2_O_3_ wrap-gate fin-array with a threshold voltage between 0 and + 1 V [15], a vertical power MISFET with fin-shaped channels[16], MOSFETs with gate recess [9, 10], or using N-Si co-doping technology [17]. Regarding the above methods, Ga_2_O_3_ epilayers grown on native substrate generally demonstrated low breakdown voltages. Elsewhere, mechanically exfoliated Cr-doped Ga_2_O_3_ substrates were utilized and transferred onto SiO_2_/Si substrate and then locally thinned to obtain E-mode MOSFETs [18]. Even though I_D_ was very low it is not feasible to mass-produce devices using exfoliation methods.

The performance of β-Ga_2_O_3_ based MOSFETs for D- and E Mode operation has been simulated by Kachhawa [19], but there are no comparable experimental results. In our work, different recessed gate etch depths were used to evaluate MOSFET electrical characteristics of heteroepitaxial Ga_2_O_3_ layers grown on sapphire substrates, for both D- to E-mode control by gate recessing. In this study, MOSFETs electrical characteristics will be discussed and compared with the previously reported simulation data [19, 20].

## Experimental

**Fig. 1:**
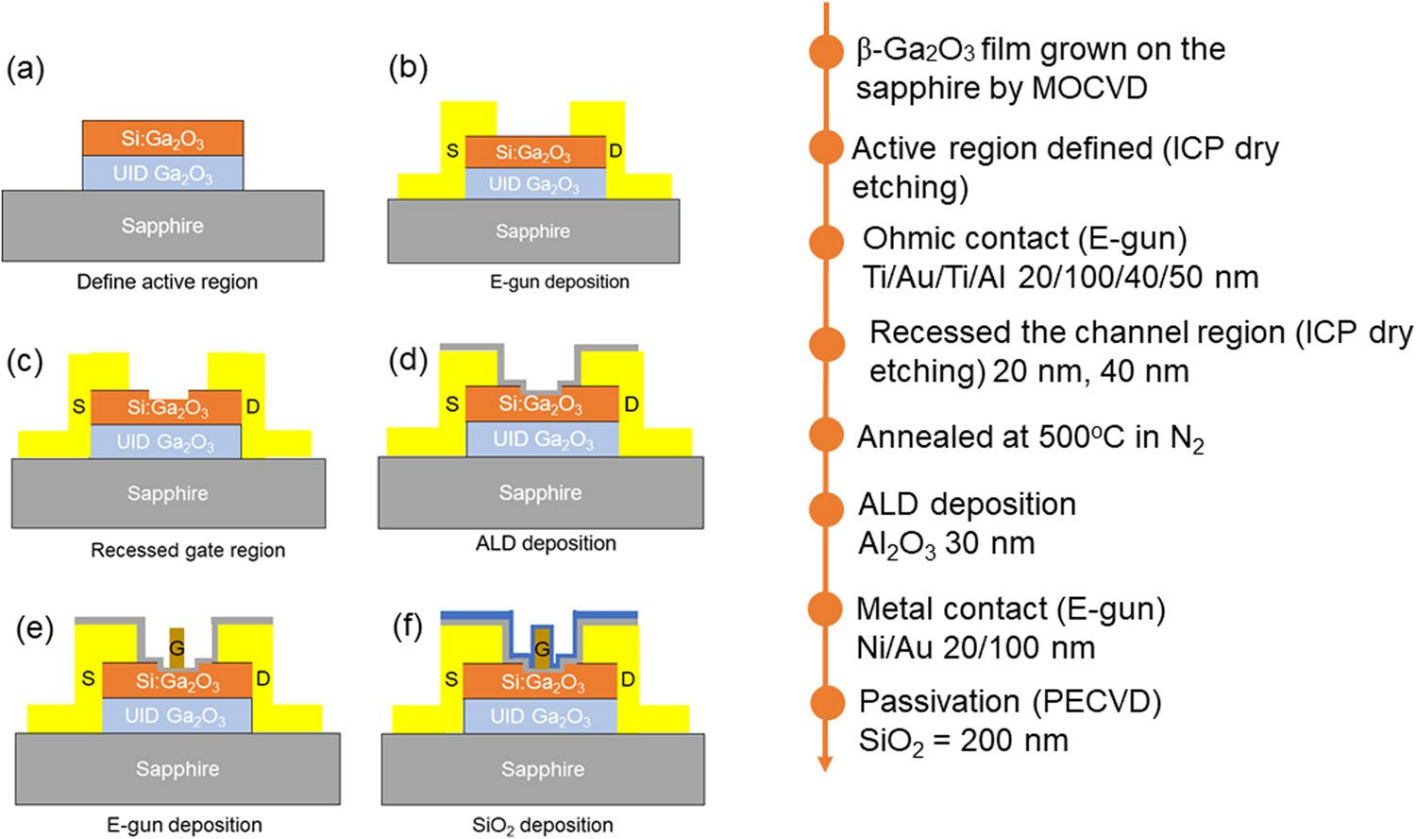
**a**The recessed MOSFET device structure and its **b**–**f** process flow

An in situ doped 45 nm epilayer and a 45 nm un-intentionally doped (UID) β-Ga_2_O_3_ layer were grown on c-plane sapphire at 875 °C by MOCVD. The Si in situ doping layer was obtained through the TEOS precursor with a 10 sccm flow rate, resulting in a measured carrier concentration (*N*_D_) of 7.2 × 10^18^ cm^−3^ and Hall mobility (μ_Hall_) 7.6 cm^2^/v-s through Hall measurement. The device active region was defined by an inductively coupled plasma reactive ion etching (ICPRIE) system using Ar and Cl_2_ to isolate each device as shown in Fig. 1(a). Following this, a Ti/Al/Ni/Au (20/100/40/50 nm) metal stack was deposited as the source (S) and drain (D) electrodes by E-beam evaporation, shown in Fig. 1 (b). To obtain an Ohmic contact for the above metals on Ga_2_O_3_, samples were annealed at 500 °C in an N_2_ ambient for 1 min. The gate region was then etched to reduce its thickness, enhance channel control as shown in Fig. 1(c). Thereafter, a 30 nm Al_2_O_3_ dielectric layer was deposited by atomic layer deposition (ALD), shown in Fig. 1 (d).

**Fig. 2:**
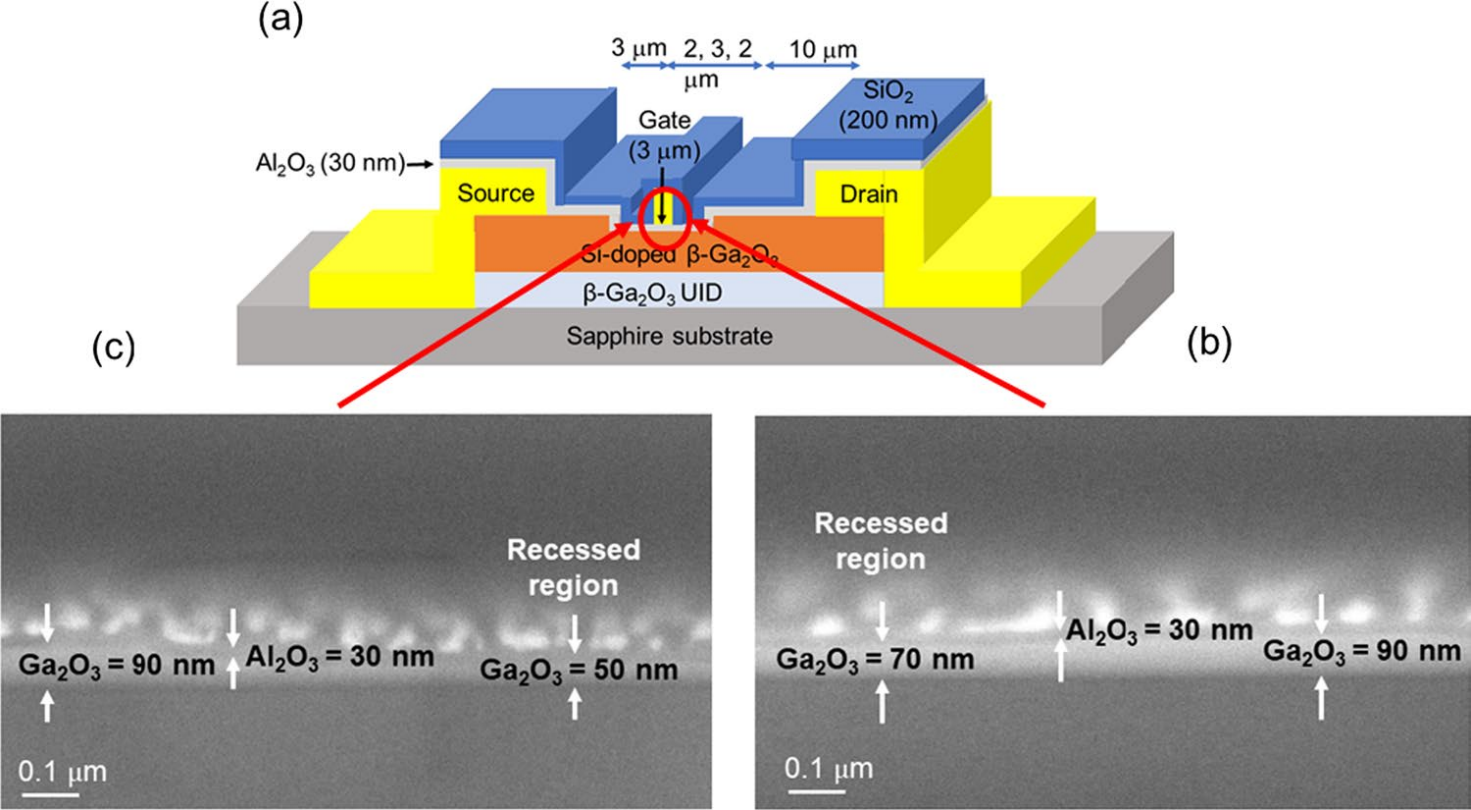
**a** The cross-sectional schematic of β-Ga_2_O_3_ MOSFET **b** SEM cross-sectional image of Gate-recessed region etching 20 nm (remained 70 nm) and **c** 40 nm (remained 50 nm)

A Ni/Au (30/150 nm) metal stack of gate (G) electrode was then deposited in the gate region by E-beam evaporation, shown in Fig. 1 (e). Finally, a 200 nm thick SiO_2_ passivation layer was grown on top and S, D and G contact pads and then are opened for measurement, shown in Fig. 1 (f). Studied MOSFETs have a gate length of 3 µm (*L*_G_), a recessed length of 7 µm, a source-gate width (*L*_SG_) of 7 µm, and a gate-to-drain width (*L*_GD_) of 10 µm. The final device schematic and etching depth were measured by scanning electron micrograph (SEM), shown in Fig. 2. Different etching depths were used to study this effect on MOSFET electrical properties. These are 20 nm, 40 nm, and a no-etch process, respectively. As β-Ga_2_O_3_ thickness before etching was 90 nm, after said etch process the remaining material thickness was 70 nm and 50 nm on sapphire substrate. The remaining material comprises a 25 nm Si-doped layer and a 5 nm Si-doped layer on 45 nm thick UID layers for 20 nm and 40 nm etched samples, respectively. After device processing, MOSFET characteristics were measured by a Keysight B1505A. Specific contact resistance (ρ_c_) and sheet resistance (*R*_sheet_) were measured by the transmission line measurements (TLMs).

## Results and discussion

**Fig. 3:**
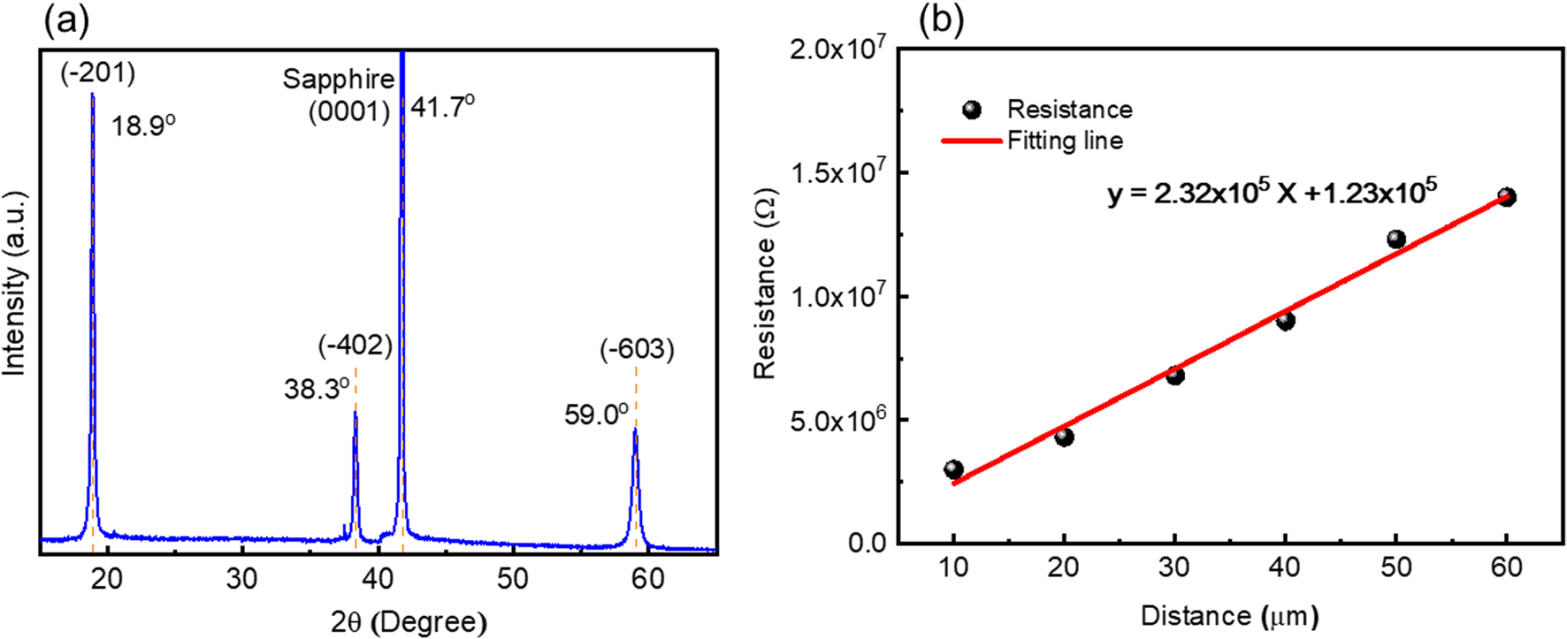
**a** X-ray diffraction pattern of the device structure and **b** TLM measurement by different distance from 10 to 60 um

The crystallinity of the device structure (n-type β-Ga_2_O_3_/ UID β-Ga_2_O_3_/sapphire) was measured by x-ray diffraction system and shown in Fig. 3(a). There existed 38.9, 18.3 and 59.0° corresponding to $\left( {\overline{2}01} \right)$, $\left( {\overline{4}02} \right)$, and $\left( {\overline{6}03} \right)$ of Ga_2_O_3_. It was worth to mention that the epilayer presented high quality single crystal even the epilayer was doped by Si with 7.2 × 10^18^ cm^−3^. Transmission line measurements (TLM) were used to evaluate Ohmic contact properties and semiconductor resistance between S and D electrodes for Si-doped Ga_2_O_3_ epilayers. The resistance as function of distance of the TLM pattern is shown in Fig. 3. The TLM was used to measure the S/D contact resistances. They are almost the same for each sample because these devices were fabricated using the same wafer. A linear relationship is clearly observed indicating that S and D metal electrodes on Si-doped Ga_2_O_3_ are Ohmic in nature. The sheet resistance (*R*_S_), transfer length (*L*_T_) and specific contact resistance (ρ_c_) can be obtained using the TLM measurement. In Fig. 3, the presented TLM results were measured from 10 µm to 60 µm. The *R*_S_ and the ρ_c_ values can be extracted using ${\text{slope}} = \frac{{R_{{\text{S}}} }}{Z}$ and $L_{{\text{T}}} = \sqrt {\frac{{\rho_{{\text{c}}} }}{{R_{{\text{S}}} }}}$. The extracted $L_{T}$ was 0.27 µm. R_S_ was found to be 46.4 MΩ/□ and ρ_c_ 3.28 Ω/mm^2^.

**Fig. 4:**
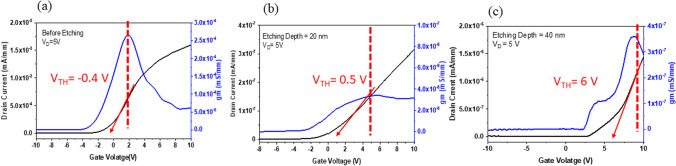
Extracting the threshold voltage by *I*_D_ − *V*_G_ measurements under *V*_GS_ = 5 V for (**a**) the non-recessed device at *V*_TH_ = − 0.4 V, **b** the device etched a depth of 20 nm at *V*_TH_ = 0.5 V, and **c** the device etched a depth of 40 nm at *V*_TH_ = 6 V

To understand the relationship between recessed depth and device operation mode, different recessed depths are considered including a non-recessed device; with depths of 20 nm and 40 nm, respectively. Figure 4 shows the relationship of *I*_D_ as function of *V*_GS_ for a Ga_2_O_3_ MOSFET with different etch depths. The threshold voltage (*V*_TH_) is defined under drain voltage condition (*V*_DS_ = 5 V) in the MOSFET linear region. The x-intercept is determined with a tangent line from the transconductance (*g*_m_) maximum point were *V*_TH_ = − 0.4 V, 0.5 V, and 6 V for non-etched, 20 nm and 40 nm etched devices, respectively. The *I*_D_ − *V*_GS_ electrical characteristic demonstrates that MOSFETs can be transferred from the depletion mode to enhancement mode. Prior to etching the MOSFET was in D-mode, but *V*_TH_ was only − 0.4 V. This could be owing to a narrow 45 nm thick Si-doped layer. Clearly, reducing the thickness of doping region, increases the resistance of the channel. For an applied *V*_G_ with either zero or negative bias, the channel was totally depleted thus the normally-on channel was turned off. Further etching resulted in *V*_TH_ increasing, yielding E-mode device operation.

**Fig. 5:**
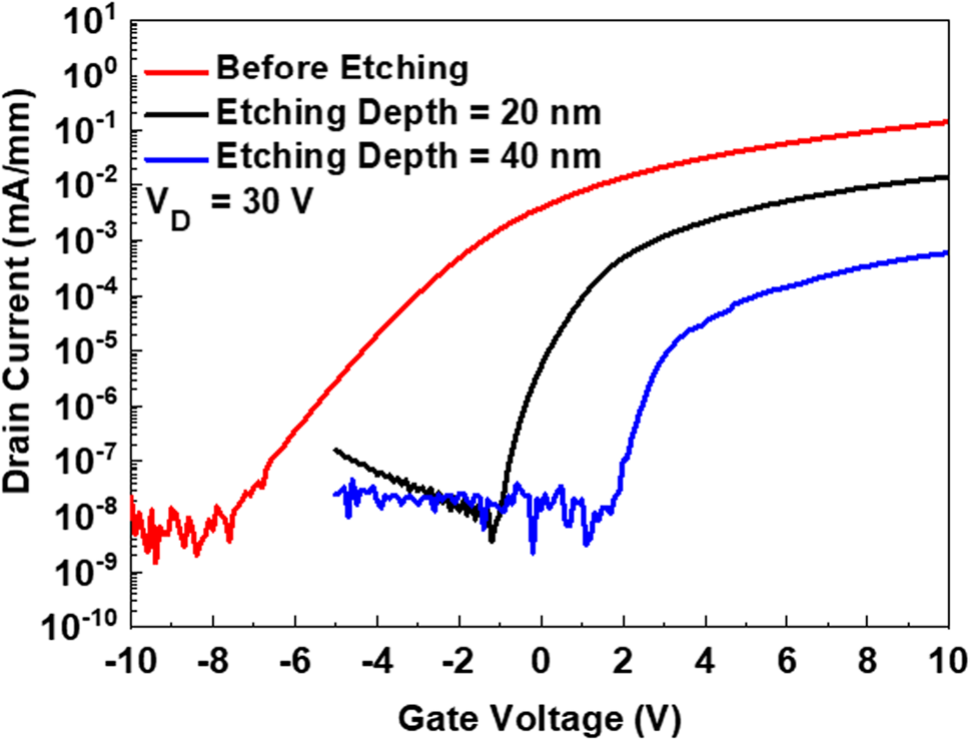
The *I*_D_s − *V*_GS_ transfer characteristic measurement for different etching depth **a** non etching **b** etching 20 nm and **c** etching 40 nm

Besides *V*_TH_, the etching depth also affected device saturation current, in this case deeper etching depths results in a lower saturation current, *I*_SAT_. In order to confirm this point, the drain current as function of gate voltage for the Ga_2_O_3_ MOSFET with different etching depths was measured for *V*_D_ = 30 V and is shown in Fig. 5. It was found that the *I*_SAT_ is approximately 140 µA/mm, 14 µA/mm, and 0.6 µA/mm for MOSFETs with a non-etched channel, and etch depths of 20 nm and 40 nm, respectively. Furthermore, *g*_m_ maximum saturation (not shown data) at *V*_DS_ = 30 V is 26 µS/mm, 2.7 µS/mm, and 0.13 µS/mm for non-etched channel, and etch depths of 20 nm and 40 nm, respectively.

**Fig. 6:**
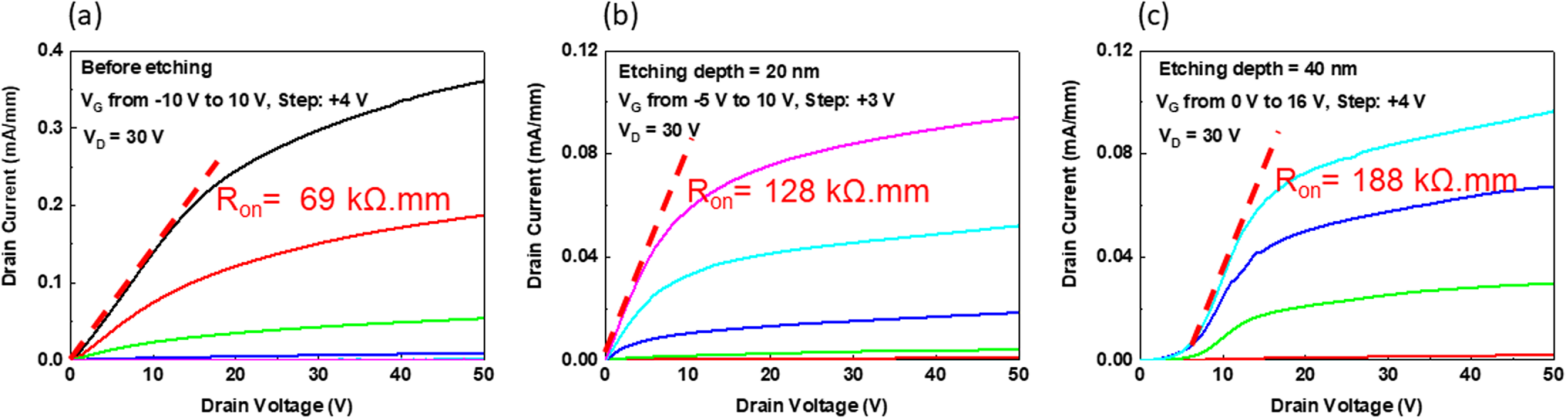
*I*_DS_ − *V*_DS_ measurement for different etching depths **a** before etching, **b** etching depth 20 nm and **c** etching depth 40 nm

Figure 6 shows *I*_DS_ − *V*_DS_ measurements for different etch depths. The V_*D*S_ ranges from 0 to 50 V in increments of 0.5 V. The maximum current shows a consistent trend with device transfer characteristic such that deeper recessed depths give lower maximum *I*_DS_ current. Likewise, turn-on resistance increased in-line with etch depth, from 69 kΩ mm, 128 kΩ mm, and 188 kΩ mm. For the singular 40 nm etched depth, under low drain bias the channel did not turn on directly. It was thought that this unusual phenomenon relates to a high channel resistance under the gate-recessed area as non-recessed devices have not experienced this problem. The contact resistances of S and D of the sample with 40 nm etching depth were the same as compared with those of sample without recess processing. The sample with 40 nm etching depth has the 50 nm channel depth (5 nm doped layer + 45 nm UID layer). Due to the channel depth being too thin, it resulted in high resistance and presented nonlinear. The device structure should be optimized for improving I_DS_.

**Fig. 7:**
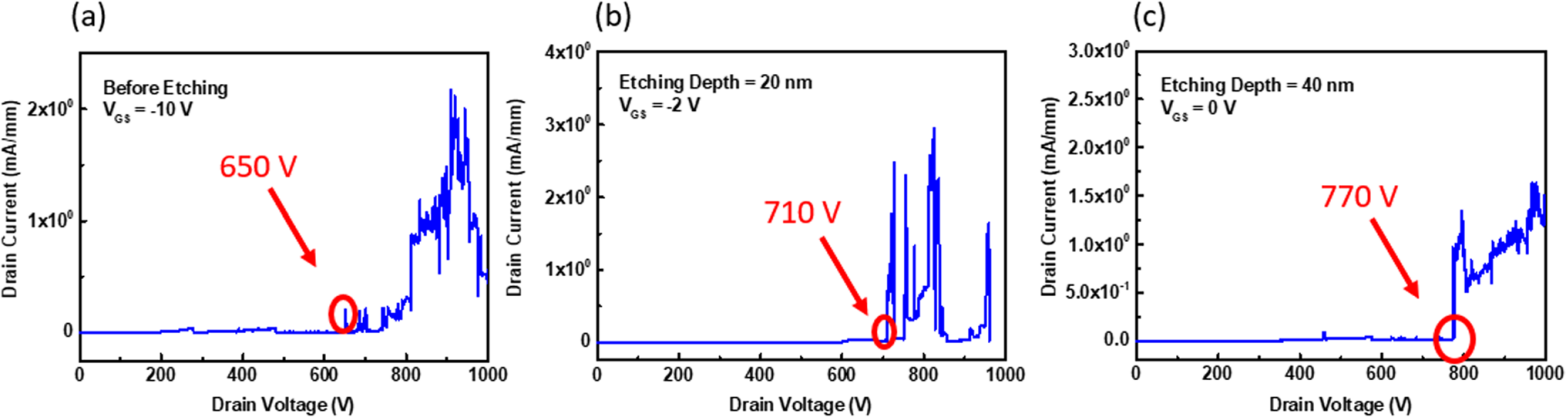
Breakdown voltage for different etching depths **a** before etcing, **b** etching depth 20 nm and **c** etching depth40 nm

It is important to evaluate MOSFET breakdown voltage for different recessed channels. The breakdown voltage characteristic of the β-Ga_2_O_3_ MOSETs for various etching depths was measured at room temperature. During measurement, these devices were operated in the OFF state. Gate voltages were held at − 5 V, − 6, and 0 V for MOSFETs without recess and with recesses of 20 and 40 nm depth, respectively. The three-terminal off-state breakdown characteristics of Ga_2_O_3_ MOSFETs with different recessed depths were measured and shown in Fig. 7. The breakdown voltage was 650 V, 710 V, and 770 V for MOSFETs without recess and with recesses of 20 nm and 40 nm, respectively. Noticeably, MOSFETs with deeper etching depths demonstrated higher breakdown voltages. One possibility for this observation is that recessed regions could present a more resistor pathway under drift condition serving to divide the high voltage from the drain. These results were similar to those reported in [9], whereby MOSFETs were homoepitaxially grown on β-Ga_2_O_3_ by MOCVD on a Fe-doped semi-insulating (010) Ga_2_O_3_ substrate [9].

Table 1 presents all electrical characteristics of Ga_2_O_3_ MOSFETs grown on sapphire substrate with different recess depths compared to those without. Obviously, MOSFETs can switch from E-mode to D-mode after recess etching; unfortunately, *I*_DS_ and *R*_on_ are scarificed. A summarizing state-of-the-art of breakdown and Ron performance for the E-mode Ga_2_O_3_ were shown in Table 2. The breakdown voltage presented the best performance in this work. In future work, the device structure will be further improved for high *I*_DS_ and low *R*_ON_.

**Table 1:** Electrical characteristic for different etching depths

Sample No	*V*_th_ (V)	Maximum current @ *V*_GS_ = 10 V, *V*_DS_ = 30 V	Breakdown voltage (V)	gm maximum (uS/mm) @*V*_DS_ = 30 V	*R*_on_ (kΩ mm)
Before etching	− 0.4	0.14 (mA/mm)	650	26	69
Etching depth:15 nm	0.5	0.014 (mA/mm)	710	2.7	128
Etching depth:40 nm	6	5.9 × 10^–4^ (mA/mm)	770	0.13	188

**Table 2:** A summarizing state-of-the-art of breakdown and *R*_on_ performance for the E-mode Ga_2_O_3_ grown on different substrates

Substrate	*R*_on_	Breakdown voltage (V)	References
Ga_2_O_3_	215 mΩ mm	198	[21]
Ga_2_O_3_	22.5 mΩ cm^2^	~ 47	[22]
Ga_2_O_3_	–	190	[9]
Ga_2_O_3_ (Vertical)	13–18 mΩ cm^2^	> 1000 V	[16]
Ga_2_O_3_ (Vertical)	135 mΩ cm^2^	263	[17]
Sapphire	47 K mΩ cm^2^	~ 155	[23]
P + − Si	35.3 MΩ cm	224	[24]
Sapphire	188 KΩ mm	770	This work

## Conclusion

Heteroepitaxial β-Ga_2_O_3_ films have been successfully grown on sapphire substrate demonstrating that MOSFETs can be switched from depletion to enhancement mode by gate-recessing. It is shown the different etching depths significantly impact device electrical performance including threshold voltage, maximum current, and breakdown voltage. In this study, the maximum breakdown voltage was 770 V and maximum current attained was 140 µA/mm. Normally β-Ga_2_O_3_ MOSFET channel regions are doped by ion-implantation, here we instigate a novel in situ doping method using TEOS during MOCVD. Further improvements will focus on increasing device saturation current and decreasing source and drain ohmic contact resistance so that this device structure and process can employed for future high-power applications.

## Data Availability

Data underlying the results presented in this paper are not publicly available at this time but may be obtained from the authors upon reasonable request.
